# Tobacco

**Published:** 1871-01

**Authors:** 


### TOBACCO.

Read before the Chicago Medical Society by F. A. Emmons, M.D., Vice-President, Dec. 12, 1870.

Three hundred and seventy-eight years ago Christoval Colon, better known as Christopher Columbus, landed upon the shores of one of the islands of the Bahama group, and found the natives using the weed which we are about to consider.

Tobacco is supposed to have taken its name from the Indian term for the pipe in which it was smoked, or Tobacco, a province in Yucatan, where it was found in great quantities.

It consists of the leaves of the *nicotiana tabacum*, an annual plant indigenous to America, but which is now cultivated in nearly all parts of the civilized world.

The leaves are large, long, and of a pale green color; the flowers are of the same color, expanding above into rose-colored segments.

The leaves, when simply dried, are destitute of the peculiar narcotic and pungent odor which is present in the prepared tobacco.

This quality is developed by moistening the dried leaves with salt-water, and heaping them together in quantities; fermentation takes place, and, during this process the nitrogenous elements of the plant are decomposed and form ammonia, which appropriates a portion of the acid in the leaves, and liberates a portion of nicotia. This last named substance, along with the ammonia, produees the characteristic odor of prepared tobacco.

There are two active principles in the weed, *nicotia* and *nicotianin.* The former is an organic volatile alkaloid; the latter a concrete volatile oil.

It is the *nicotia* mainly on which the characteristic effects of the plant depend. Nicotia was obtained in its pure state about the year 1827, by a number of gentlemen, among whom were Messrs. Reimann and Passelt.

They obtained it by distilling the leaves with potassa or soda.

It is a colorless, transparent liquid, free from oxygen, but it contains a large proportion of nitrogen, it being composed of two equivalents of nitrogen, twenty of carbon, and fourteen of hydrogen.

Cigar-smoking was introduced into Spain at an early date, the custom having been brought over by companions of Columbus, who had acquired the habit from the natives of the West India Islands on their discovery in 1492.

John Nicot, (from whom the nicotia takes its name), Sir Francis Drake, and Sir Walter Raleigh were more particularly influential in introducing tobacco into Europe in the latter part of the sixteenth century, and, by their efforts, it was more prominently brought before the crowned heads, who encouraged its culture, and soon became addicted to its use.

This habit spread throughout the continent among all classes. Its cultivation has increased until it is a staple product of the country, and the demand for it is nearly equal to that of coffee and sugar, and it is apparently as indispensable, as is evinced by the orders of the war and navy departments of our own nation and of England, it being issued with the rations of subsistence.

As a remedial agent, tobacco has been used with considerable success in a number of diseases, among which the following are the more prominent: Nervous cough, whooping-cough, spasmodic cough, epilepsy, dropsy, intussusception, spermatorrhœa, constipation, strangulated hernia, and tetanus.

In the treatment of *tetanic spasm*, this remedy is considered second to none at our command, unless it be opium and *calabar bean.*

In strangulated hernia, there appears to be no remedy better adapted to a thorough relaxation of the system, permitting an easy reduction of the tumor after other remedies have signally failed.

It is undoubtedly a valuable agent in constipation when there is a torpid condition of the bowels, superinduced by a loss of the secretory power of the glands.

It has its advocates in consumption, asthma, and all forms of spasmodic cough, neuralgia, spermatorrhoæ, epilepsy, and various nervous diseases.

Experiments upon animals go to prove that its *active principle*, when administered internally or endermically applied, acts as a virulent poison, producing death in a very few moments.

Primarily in its action, it lowers the circulation, quickens the respiration, dilates the pupil, and excites the muscular system. Ultimately, the pupils become contracted, the respiration slow and labored, with trembling of the limbs, and faintness, retching, vomiting, convulsions, and, finally, paralysis of the brain and motor nerves.

When it is gradually administered, there is general debility and exhaustion, both of animal and organic life, loss of hair and the venereal powers, sloughing of the eyelids, and total blindness.

After death the blood is found to be deficient in fibrin and red globules.

The mucous membrane of the mouth, nose, and trachea are found softer, more tumid and vascular than usual.

The action of *nicotia* upon the human subject is not unlike that upon the inferior animals. In small quantities, it produces irritation of the fauces, and œsophagus, with abundant salivation; there is a general excitement of the brain, and nervous system, with headache, burning sensation on the tongue, and in the stomach, which soon penetrates the entire system. The mind becomes dull, and the individnal remains in a drowsy, listless condition, with a sense of heaviness in the head. There is indistinct vision, and dilitation of the pupils, the eyes being peculiarly sensitive to the light.

The hearing becomes affected and the respiration frequent and laborious, with a feeling of oppression, and accompanied by dryness of the throat.

As the effect increases, signs of debility and exhaustion become more marked; the head cannot longer be held erect; the face assumes a death-like palor; the features are relaxed, and the extremities become cold—the chill gradually extends to the body.

These symptoms are followed by complete loss of consciousness; the respiration is now short, hurried, and incomplete, accompanied by trembling of the limbs; marked rigors, and severe spasms; the pupils become contracted and the sphincters relaxed.

The symptoms become alarming, and death may supervene by paralysis of the respiratory nerves.

Discontinue it, and there is a subsidence of the symptons, and a gradual return to health.

Let us turn our attention for a few moments to the consideration of the effects of tobacco upon the system in a more general sense.

It must be admitted that tobacco, employed in any manner, has a powerful effect upon the nervous system. The effect is regulated by the amount used, the method of introduction, and by the peculiar idiosyncracy of the individual.

Persons with an out-door or purely physical employment, are not so visibly affected by its use; indeed, it is often positively beneficial, when used with moderation, to persons exposed to a malarious or contagious atmosphere.

But in individuals of slender form and sedentary habits, and those of a nervous temperament, especially in early life, the effect is very marked, with an undue nervous excitement, sleeplessness, loss of flesh, and general debility. The effect is, no doubt, produced by its influence upon the system, the brain, or more particularly, the *medulla oblongata* and motor nerves, and manifests itself through the nerves of respiration and the sympathetic system, producing all the long train of symptoms found in those persons addicted to its extravagant use.

The influence of tobacco upon the system in the quantities and manner generally employed diminishes the amount of feces, lessens the amount of urea and chlorine, and *increases* the amount of free acid, uric acid, phosphoric and sulphuric acids, eliminated through the kidneys.

The sedative effect of tobacco upon the nervous system, and the general prostration produced by its use, is counteracted in a great degree by resorting to stimulants; hence the youth just ripening into manhood, commencing the use of the weed in order (as he thinks) to make himself manly, frequently indulges for the first time in the use of alcoholic stimulus, to counteract the effect of the tobacco, and it thereby often becomes a source of intemperance.

Yet, however much we may be opposed to intemperance, no doubt the use of alcoholic stimulus in some form should be encouraged in those persons addicted to the extravagant use of tobacco, if unable or unwilling to discontinue it—for alcohol, undoubtedly modifies the deleterious effect of tobacco upon the nervous system.

To my mind, some national characteristics are undoubtedly produced by the peculiar manner of its employment.

We are a nation of tobacco-chewers from early youth to old age; the *quid* finds its abiding place in the mouth of the American, and the attendant evils of every form of dyspepsia are plainly traceable to its use.

But if chewing is specially an American custom, so equally is smoking a German characteristic, and its effects are quite as plainly observable.

If chewing superinduces dyspepsia, and has, to a degree, rendered us a nation of dyspeptics, the abundant use of the cigar and pipe, and the studious habits of the Germans have made them a glass-wearing people.

The German student pursues his labors and whiles away his hours of recreation in a cloud of tobacco-smoke and finds his eyes need *aids*, which even spectacles too often fail to afford.

In conclusion, then, considering that the evil effects of an habitual or excessive use of tobacco *far outweigh the benefits which confessedly appertain to its moderate consumption under favorable and exceptional circumstances*, I would ask if it does not behoove us, as the medical advisers of the people, to discountenance the use (excepting only as a therapeutic agent) of an article whose active principle is second only to *prussic acid* in its rapidly destructive effect upon organic life.

